# Medical News and Miscellany

**Published:** 1898-03

**Authors:** 


### Medical News and Miscellany.

**Dr. W. H. Lancaster** has removed from Waco to Flatonia, Texas.

---

**Dr. C. S. Bobo** has changed his location from Boyd to Weatherford.

---

**Dr. E. E. Guinn,** formerly of Alto, has removed to Jacksonville.

---

**Dr. D. S. Reid** has removed from Wortham to Palestine, where he has formed a copartnership with Dr. J. H. Evans.

---

**Dr. S. P. Rice,** of Marlin, is in Chicago, and will spend some months there in attendance at the Post-Graduate School.

---

**Dr. Clay Johnson,** of Corsicana, was married to Miss Alice Jester, daughter of Lieutenant Governor Jester, on February 16th.

---

**Dr. Edward B. Jackson,** of Houston, Texas, desires a young physician partner. Must be sober and energetic. Write or call for particulars.

---

**Dr. R. Harvey Reed,** of Rock Springs, Wyoming, has been appointed Surgeon General of the National Guard of that State, with rank of colonel.

---

**Married.**—At Kerrville, the home of the bride, February 1st, ult., Dr. L. W. Cock, of San Marcos, Texas, to Mrs. Sallie Lett, formerly of LaGrange, Texas. No cards; no cakes.

---

**The Texas State Medical Association** will meet in

Houston on April 26th, and remain in session four days. From present indications the attendance will be very large. Houston is an excellent place for the meeting, being well provided with railroad and hotel accommodations, and the medical profession of the Bayou City will no doubt entertain in their usual royal style.

---

**Dr. Nicholas Senn,** of Chicago, was lately taken to Galena, in care of a sheriff, to answer to the charge of contempt of court. As it turned out that his evidence was immaterial to the case, where he refused to appear, and as the judge found out he was a very busy man, having much more important duties than the neglected one pressing upon him, he was forgiven by the court.—*New York Medical Record.*

---

**Uterine Inertia.**—Dr. W. B. Dorsett has used phosphate of strychnine (grain $\frac{1}{100}$, gradually increased to grain $\frac{1}{25}$) with very satisfactory results during the gestation period of anemic and debilitated women. In addition to the local action upon the uterine musculature, it promotes assimilation, relieves constipation and prevents the often observed chilliness or rigors after labor. Under this treatment he has not found it necessary to employ ergot for the past five years in his obstetric practice.—*Denver Medical Times.*

---

**Meeting of District Medical Examiners of Texas.**—A call has been issued by the board from the 13th judicial district for a State meeting to be held in Corsicana on April 11th, prox. The purpose of the meeting, we understand, is mainly to formulate a uniform system of examinations for applicants for license to practice medicine. This being its purpose, it occurs to us that the meeting should be held at the time and place where the largest attendance can be had. We endorse the suggestion made by our neighbor, the *Texas Medical News*, that the meeting be held in Houston during, or before, the session of the Texas State Medical Association.

---

**The American Medical Association** will hold its next meeting in Denver on June 7th, 8th, 9th and 10th. This will be an unusually good time for the doctors of Texas to visit Colorado and get a view of its magnificent scenery and inhale some of the pure mountain ozone. Our physicians should learn more of the climate of Colorado, since it is the very best place in the world for a large class of our cases affected with lung trouble. To be properly appreciated as a health resort it is necessary, so our neighbors of Colorado say, to see their wonderful mountain scenery, but more necessary, in fact, absolutely essential, to breathe their pure, light, health-giving air. The Fort Worth and Denver City Railway is very anxious for Texas doctors to see for themselves what the climate of Colorado is, and they propose to put on the most liberal rates for the coming meeting. Let us try to get up a good representation, and where possible, take the good wife and let her have some of this "famous ozone." Besides the enjoyment of the meeting and the advantages of the trip to Colorado, we are to have an excursion to Salt Lake City. To many of our Texas people this will be one of the greatest attractions.

### Death to Corns.

Under "Death to Corns," the *National Druggist*, November, 1897, gives the following formula:

| | |
|---|---|
| Ex. of Cannabis Indica | 1 part |
| Salicylic acid | 10 parts |
| Oil of turpentine | 5 parts |
| Glacial acetic acid | 2 parts |
| Cocaine (alkaloidal) | 2 parts |
| Collodoin qs. to make | 100 parts |

Mix; apply a thin coating every night, putting each coating on top of the preceding one, until finally the whole drops off, bringing the indurated portion and frequently the whole corn with it.—*Western Medical and Surgical Gazette.*

**Carbolic Acid Gangrene.**—The danger of prolonged topical application of even the weakest solutions of carbolic acid is well known to the medical profession. Since it is a household remedy, the editor of the *New York Medical Journal* (September 11, 1897) thinks the danger of its use should be made more extensively known to the laity. Dr. J. Levai, of Buda Pest, reports twenty-six cases of carbolic acid gangrene out of a total of 20,417 surgical cases. In nearly every case the acid was used in the form of a continuous application without medical advice. In some cases mummification of the soft parts resulted, but generally gangrene of the whole or part of a finger through its entire thickness. Morestin (*Centralblatt fur Chirurgie*, August 14, 1897) reports a case of mummification of the finger of a child, also exarticulation at the junction of the first and second phalanges after the application for twenty-four hours, by the mother, of what was apparently a very weak solution of carbolic acid. By experiment Levai confirmed his clinical experience, showing that the acid was the real cause of the trouble.—*Western Medical and Surgical Gazette.*

